# National Hospital for the Paralysed and Epileptic

**Published:** 1901-12-21

**Authors:** 


### The Institutional Workshop.

### NATIONAL HOSPITAL FOR THE PARALYSED AND EPILEPTIC.

### REORGANISATION COMMITTEE'S REPORT AND RULES ADOPTED.

Mr. Burford Rawlings' Pension.

The protracted controversy regarding the management of the National Hospital for the Relief and Cure of the Paralysed and Epileptic, would seem at length to have been settled by the adoption by the governors of the new set of rules (based on the recommendations of Sir Edward Fry's Committee of Inquiry) submitted by the committee of re-organisation, of which Mr. Melvill Green was chairman, and the pensioning of Mr. Burford Rawlings, the late secretary and general director, around whose position the dispute mainly centred.

A special general meeting of the governors of the National Hospital was held on Thursday, 12th inst., "To consider the report of the committee appointed by virtue of the resolutions passed at the special general meeting of governors and subscribers, held on July 6th, 1901, and the proposed new rules for the constitution of the hospital referred to in such report, and to pass such resolutions as may be thought expedient for the purpose of repealing, altering, or adding to the now existing rules, and (or) adopting (whether with or without modifications) the proposed new rules, and (or) dealing with the recommendations contained in the said report.

The Hon. Sydney Holland, who was voted to the chair, said that Mr. Burford Rawlings had consulted him as to whether he should come to that meeting, and was anxious that his absence should not be misunderstood. He was sure they would all appreciate that, under the circumstances, that absence was dictated by good taste. He thought also that they would agree that the discussion should be confined to the future constitution of the hospital, and that no references should be made to the unfortunate differences in the past.

The report of the committee of reorganisation which consisted of Messrs. Melvill Green, Timothy Holmes, John Danvers Power, and James Wigan, nominated by the committee of March 23rd, 1901; the Hon. Sydney Holland, Messrs. Colin F. Campbell and John Pearman, nominated by the board of management; Sir Felix Semon and Dr. Joseph Arderne Ormerod, nominated by the medical staff, stated:—

We have carefully considered the existing rules and bye-laws of the Hospital in their relation to the report of Sir Edward Fry's Committee of Inquiry which you have adopted. In redrafting the rules for your approval we have in the main confined ourselves to the recommendations of the report by which we have felt bound.

We enclose herewith the draft of the new rules which we recommend for adoption in the place of the existing rules. We advise that the existing bye-laws be abolished *en bloc*, and that the Board of Management (all the members of which, it will be observed, must be elected on the coming into force of the new rules) be left to make such bye-laws as they may from time to time find to be necessary. We are not able to advise the governors to go quite so far as the Committee of Inquiry recommended in paragraph 106 of their report, which suggested that new bye-laws should not be effective until confirmation by a general meeting. By rule 39 in the enclosed draft, we have provided that new bye-laws shall be immediately effective, but that unless they are confirmed by the following annual general meeting, they shall be annulled. We think that this safeguard will be sufficient, inasmuch as the rules themselves have, we trust, been made to cover all fundamental matters affecting the constitution of the hospital, and it may be desirable to make a bye-law effective without delay.

We have given anxious consideration to Paragraph 77 of the Report of the Committee of Inquiry, which was as follows:

"It should be added in justice to Mr. Rawlings that the course he pursued in the case of this child, and in most of, or perhaps all, his dealings with the Medical Staff, received the approval and support of the Board of Management; that, in our opinion, he was throughout acting in the manner which he thought to be his duty, and which was in the spirit of the instructions which he received from the Board; and that on them rather than on him rests the responsibility for the attitude he assumed towards the Medical Staff. We cannot refrain from expressing our hope that in any change which may result from this Report every consideration will be shown to the just claims of Mr. Rawlings on the gratitude of the Institution."

We have already mentioned that, under the proposed new rules, a new Board of Management will be elected as soon as may be. It therefore only remains for us to recommend what, in our opinion, should be the manner in which Mr. Burford Rawlings' just claims on the gratitude of the institution should be met. Mr. Rawlings has been in the service of the hospital for 35 years, and during the greater part of that long period his work was carried out without any friction. During the whole time the hospital has been indebted to him for his untiring and most successful exertions in raising money for the current expenses, the new buildings, and the convalescent hom e. To Mr. Rawlings is due, in a very large measure, the fact that the hospital is what it is, and his labours have far exceeded anything that could reasonably be expected of a hospital secretary. His present salary amounts to £600 per annum.

We need not enumerate all the reasons which have influenced us in making our recommendation. After full consideration of all the circumstances, including the fact that the funds of the hospital have been subscribed primarily for the relief of the sick poor and that incidental expenditure must be carefully watched, we have come to the conclusion that a pension, to be paid during the pleasure of the governors, of £450 per annum will be a suitable provision.

This was signed by all the members of the committee, but the following minority report was appended:—

The undersigned think that, having regard to the great services which Mr. Burford Rawlings has rendered to the hospital during the past 35 years, the proposed pension of £450 is insufficient and should be increased. (Signed) Colin F. Campbell, Sydney Holland, John Pearman.

With reference to this Report, the Board of Management issued to the governors on the 3rd instant a circular drawing attention to the points in Sir Edward Fry's report favourable to the management, and continuing:—

The Board think it right to bring to the attention of the governors certain proposals which, in their judgment, must be injurious to the future working of the hospital. The most serious is the proposal to divide the work of carrying on the daily administration of the hospital between three co-ordinate independent authorities. Rule 41 provides for the appointment of a secretary, a senior house physician, and a lady superintendent, in "sole control of their respective departments under the Board." The duties and functions of these three officers must necessarily overlap, and in the absence of some authority to whom instant and effective reference and appeal can be made upon matters of daily and hourly occurrence, as to which differences of opinion may arise, confusion must ensue, with consequent injury to the interests of the patients and of the institution. The Board feel strongly that there should be some one person responsible to the Board for the daily conduct of affairs.

The Board also call the attention of the governors to certain other proposed alterations. By proposed Rule 10 every member of the medical staff will upon election immediately become a governor. In the opinion of the Board seven years' previous service upon the active staff should be necessary to qualify for this privilege. The Board also consider that the quorum required for a general meeting (Rule 22) should be independent of governors who are members of the medical staff, and that provision should be made in Rule 31 that the 10 ordinary members of the Board shall not be members of the medical profession.

The Board have considered the recommendation as to the pension to be allotted to Mr. Burford Rawlings, and are of opinion that the amount recommended by the committee is insufficient and should be increased.

The Board are of opinion that the case is exceptional. The salary paid has not been large, when the duties and responsibilities with which Mr. Rawlings has been entrusted are considered, together with the length and ungrudging nature of his service. During the 35 years since his appointment he has not missed attendance at a single meeting of the governors, Board, or House Committee. In addition, Mr. Rawlings has taken upon himself labours involving large demands upon his own time, in respect of which he has received no remuneration. These include, amongst other undertakings of permanent value, the work, financial and other, comprised in the rebuilding of the hospital and its convalescent home at a cost of £115,000. During his tenure of office the invested property of the hospital in buildings and other securities has increased from £7,500 to £225,000; the beds occupied by patients from 28 to 200, the endowed pensions for the incurable from 2 to 90, and the reliable income from £750 to £10,500. The board are of opinion that the funds of the charity are properly chargeable with adequate remuneration for those in its service, and that the proposed pension would not adequately give effect to the view of the Committee of Inquiry that every consideration should be shown to the just claims of Mr. Rawlings on the gratitude of the institution nor would it discharge the material obligation of the institution to one whose service practically extends to its whole history and who has identified himself with every feature of its life.

Mr. Melvill Green moved the adoption of the Reorganisation Committee's report, so far as it related to the rules. He thought the rules as now arranged would work well in the future. They had taken care to provide for the representation of the medical staff on the committee, and the abolition of the office of director, which had been at the bottom of all the friction. They took the precaution, after they had finished their work, to send a copy of the rules to every member of the Board, and waited a fortnight to see if anyone thought them unworkable; but until they received the circular, signed by Mr. Russell, they had no idea they would not be agreed to. He was sorry they had received that circular, and sorry to have to refer to the past, but that circular had given him a worse impression of the capacity of the Board of Management than almost anything that had gone before. Mr. Green was proceeding to criticise and characterise the attitude of the authors of the circular, when the chairman appealed to him to omit all references to the past, and to confine himself to answering the arguments in the circular. Mr. Green then proceeded to criticise the clauses of the circular, especially objecting to the proposals to fetter the representation of the medical staff.

Sir Jas. Crichton Browne seconded the motion, and expressed the strongest objection to the proposals of the Board, which he declared were an insult to the medical profession.

Mr. Melvill Green then went through a number of verbal alterations to various rules, which were adopted.

Rule 41 read:—There shall be a secretary, a senior house physician, and a lady superintendent, who shall have the sole control of their respective departments under the Board, and whose duties shall be defined by the Board and embodied in the by-laws. It shall be their duty to report to the Board, and in all cases of difficulty to consult, so far as possible, the chairman, or failing him the vice-chairman, and to act in accordance with his advice.

And when this was reached,

Sir Henry Burdett proposed, in order that the recommendation of Sir Edward Fry's committee should be given real effect, to insert after Senior House Physician, "who shall be appointed resident medical superintendent and shall be the responsible medical head of the whole establishment under the board of management." Having regard to the nature of the maladies treated, and that they had patients of long residence, he thought it of the first importance that there should be a medical head of the institution and that he should be responsible. He felt that they as governors, desirous of bringing about a final, ultimate, and permanent decision, should adopt the inquiry committee's recommendation in its entirety.

Mr. Bennett seconded this amendment.

Dr. Bastian said University College Hospital, with which he had been connected for 30 years, had a resident medical officer, a sister superior, and a secretary, and he had never heard of any difficulty arising. But he thought it would be a mistake to have a permanent resident medical officer. It would be better to have one elected for two or three years—subject to re-election. There would be no difficulty in getting hold of suitable men for the post.

Mr. Melvill Green hoped the rule as it stood would not be altered in a syllable. That rule had received twice as much attention from the committee as any other, and they were sure this was the best practical arrangement.

Sir Felix Semon, as a member of the staff, said he thought the rule as it stood perfectly satisfactory, and should have a fair trial.

The amendment was put and lost.

After some further discussion and the adoption of verbal alterations, which completed the consideration of the rules in detail,

Mr. G. W. E. Russell rose to move a direct negative to the committee's proposals, holding strongly that no case had been made out for the reconstitution of the hospital. He referred to the circular issued by the Board and said that though he had signed it as chairman of the meeting, he did not draft it or contribute one word to it, and it was in no sense due to him. He would not be understood to protest against its issue by the Board; and, on the other hand, neither the Board nor Mr. Bower nor Mr. Rawlings were associated with him in his attitude at that meeting.

The resolution was then put to the meeting and carried, only two hands being held up against it, and a demand for a poll, not being supported by the requisite number, was lost.

The Chairman then submitted the second part of the report recommending a pension to Mr. Burford Rawlings. They would note that £450 was the amount put forward, but there was also a minority who thought £450 was not enough. It would be difficult in a meeting like that to come to a decision, but he thought the simplest method of procedure would be for Mr. Melvill Green to move the report recommending £450 and then for there to be a general discussion, with suggestions rather than separate amendments; and he should here ask the meeting to accept the sum supported by the largest number of votes, even though that might be in a minority of the whole meeting, as, of course, any one sum might be.

Mr. Melvill Green then moved that a pension of £450 be given to Mr. Rawlings. He knew there was a difference of opinion, but he hoped that sum would be accepted as a compromise. Mr. Rawlings had been secretary for 35 years, and for the last eight or nine years his salary had been £600. They must not forget that he was made director by the action of the Board, and all that had gone as they could wish it had not gone, had belonged to the office of director. As secretary he had been a most excellent secretary. But they could not now have him go on with the secretaryship with the directorship cut off. So it became a question what pension was due to their secretary whose office was abolished. He could not be blamed for accepting the power that was offered to him; the blame lay with the Board for creating the office and for extending the power and allowing it to go beyond reasonable limits. And he did not think the blame stopped at the Board; the Governors, especially in London, knew that that was spoken of as the "one-man hospital." Mr. Rawlings was much to be pitied for the result. His whole heart and his whole life were bound up in the hospital, and it was very sad that he should have to be removed.

Mr. Wigan seconded the resolution.

Mr. Nelson said he did not know Mr. Rawlings personally, but he had heard a great deal of his services, and, as far as he could gather, the testimony was all of the same kind, that he was a man who had devoted his life to the work he had in hand, and had never been adequately paid for what he had done or for the kind of man he was. He therefore thought that the pension should be £600.

Mr. Smith objected to the funds subscribed being spent in this way at all. He had never heard that Mr. Rawlings was paralytic.

Sir John Paget inquired what would be the pension granted in a Government office, and was told two-thirds of the salary.

Mr. Pearman suggested as a compromise that they should add a year's salary to the pension.

Sir Henry Burdett supported this suggestion. They could not conduct great charitable institutions if the men who devoted their lives to their financial success were not properly treated. Outside that hospital there was no one more respected than Mr. Rawlings. He had a unique knowledge of hospital matters, and the result of his work had been to add largely to the volume of subscriptions to all hospitals. When ill or past work all should be able to look to their work to provide a suitable retiring allowance. Mr. Rawlings had so given himself up to the work of the hospital that he had no time or thought for himself, and he had been underpaid and overworked for many years past. Hence he deserved and needed a liberal pension. He thought the proposal to add a year's salary to the pension was distinctly a wise one, and should unite the governors in its support.

Sir John Paget said he thought even the £450 was too much; they should be just before they were generous.

The Chairman said if Sir John's ideas were to prevail they would never get good men to do the work of the hospitals. Personally, he thought if he secured Mr. Rawlings' services for a hospital they would be worth £20,000 a year in new revenue. They must remember he had thought he was doing right. He had had hard knocks and had gone under, and after years of the most devoted service the remainder of his life would be very bitter. He had never known any secretary of any hospital who had done so much, and he thought they should have the grace to add to the pension a year's salary as a present. In all sorts of ways he had done extra work, on building funds, in special begging years, and so on, and had never had extra pay. Let them consider this in the light of a recompense for that extra work. He put it to the meeting that to the recommendation in the committee's report should be added the words, "And that a present be made to Mr. Rawlings of one year's salary in recognition of past special services."

This was carried by 25 (or as another count made it 28) to 12, and the substantive motion was carried *nem. con.*

A vote of thanks to the Chairman closed the proceedings.

